# Recent medical technologies guidance relevant to surgeons

**DOI:** 10.1308/rcsann.2012.94.7.525

**Published:** 2012-10

**Authors:** Bruce Campbell

**Affiliations:** Chair, Interventional Procedures Advisory Committee; Chair, Medical Technologies Advisory Committee; National Institute for Health and Clinical Excellence, MidCity Place, 71 High Holborn, London WC1V 6NA. www.nice.org.uk

In a previous issue of the *Annals*, I described the medical technologies evaluation programme that was established in 2009 and which has matured since that time.^1^ This NICE programme selects and evaluates devices and diagnostics notified by manufacturers and produces guidance on them, based on claims of advantages to patients and/or to the health service. Evaluation demands clear value propositions but it is often done on the basis of rather limited published clinical evidence.^2–3^ The advice and opinions of clinical experts is a very important part of the evidence used by the advisory committee.

Medical technologies guidance relates to specific products but it may help to steer clinicians and the NHS towards adopting general types of technology, provided that the evidence base for other similar devices is adequate. Published medical technologies guidance relevant to surgical practice includes the following.

## Inditherm patient warming mattress for the prevention of inadvertent hypothermia (MTG 7)

This is a pressure-relieving system of mattresses and blankets, which incorporates a uniform direct heating surface, with safety features to prevent overheating. The mattress can be left on the operating table and cleaned between patients, just like a normal theatre table mattress. Guidance recommends that the Inditherm system should be considered as an alternative to forced air warming, which is one component of the current standard of care for preventing perioperative hypothermia.^4^ There may be circumstances in which the two types of warming are used together. The evidence supports the claim by the manufacturers that the Inditherm mattress is as effective as forced air warming in maintaining core body temperature above 360 degrees but Inditherm may have practical advantages. Cost modelling, including the costs of equipment, disposables and projections of likely reduction of postoperative infections as a result of avoiding hypothermia, predicted a potential annual cost saving of £9,800 per operating theatre if Inditherm is routinely adopted instead of forced air warming.

It is worth clarifying the relationship between medical technologies guidance and NICE clinical guidelines (which recommended forced air warming and not warming mattresses, based on the evidence available when GC65 was prepared).^4^ Medical technologies guidance may offer recommendations on new, alternative technologies, with explicit information about their potential advantages and likely cost consequences. It introduces considerations, based on novel and bespoke evaluation methods, which are new and additional to any clinical guideline: the latter may be updated to incorporate the more recent evidence and recommendations made for medical technologies.

## The VeriQ system for assessing graft flow during coronary artery bypass grafting (CABG) (MTG 8).

This is a Doppler ultrasound system, incorporating reusable sterile probes, for measuring flow in bypass grafts. It was clear to the Committee that practices vary and opinions differ about techniques for checking graft flow during CABG operations. However, thorough examination of the evidence presented did support the claim that using VeriQ (either alone or as an adjunct to other methods of checking bypass graft function) would prompt revision of imperfections in a small proportion of CABGs, which could otherwise lead to occlusion. Although the numbers of patients who might suffer serious clinical deterioration as a result would be small, the damage to them and the costs of their care supported the case for adopting VeriQ, with an expectation of saving £115 per patient, assuming its widespread and regular use.

## moorLD12-B1: a laser Doppler blood flow imager for burn wound management (MTG 2)

This device is an adjunct to experienced clinical evaluation of the depth of burns and the need for skin grafting. There was evidence that this device can usefully guide treatment decisions when there is uncertainty about burn depth: it allows earlier planning and avoidance of needless skin grafting in some patients. Cost modelling suggested savings per patient scanned of around £1,240, based on a 17% reduction in the number of skin grafting operations.

## The PleurX® peritoneal catheter drainage system for vacuum-assisted drainage of treatment-resistant, recurrent malignant ascites (MTG 9)

This system comprises a silicone catheter and 1-litre vacuum bottle with associated kit that can be used in patients’ homes instead of traditional inpatient paracentesis for distressing recurrent malignant ascites. There is good evidence that the system is effective and has a low complication rate. It has the potential to allow earlier treatment in the community when ascites starts to cause distress, instead of waiting for large-volume paracentesis in hospital, associated with an average inpatient stay of almost three days. Using the PleurX® system offers not only the opportunity to improve quality of life for patients with advanced malignant disease but it is also associated with a likely cost saving to the NHS of some £679 per patient.

## The MIST therapy system for the promotion of wound healing (MTG 5)

This technology involves the delivery of ultrasound energy through a saline mist. It is claimed to enhance healing of chronic ‘hard-to-heal’ wounds. Based on the evidence available, MIST appeared to have real promise but there was insufficient evidence about just how effective it is in clinical practice. An explicit recommendation was therefore made for research and NICE used new capacity that it has created to facilitate the setting up of collaborative research between manufacturers and the NHS to answer the outstanding questions. That research has now been commissioned and MIST will be reviewed for updated medical technologies guidance when the results of that research are available.

## Other guidance relevant to surgical practice

Between November 2011 and June 2012, NICE published further guidance documents (or reviews of existing guidance) that are of relevance to surgeons. Please refer to the NICE website (www.nice.org.uk) for further details.

## Clinical guidelines

> Colorectal cancer – www.nice.org.uk/CG131
> Organ donation – www.nice.org.uk/CG135
> Upper GI bleeding – www.nice.org.uk/CG141

## Interventional procedures guidance

> Balloon dilatation of the Eustachian tube – www.nice.org.uk/IPG409
> Closure of anorectal fistula using a suturable bioprosthetic plug – www.nice.org.uk/IPG410
> Endoscopic transluminal pancreatic necrosectomy – www.nice.org.uk/IPG411
> Single-port laparoscopic nephrectomy – www.nice.org.uk/IPG414
> Breast reconstruction using lipomodelling after breast cancer treatment – www.nice.org.uk/IPG417
> Incisionless otoplasty – www.nice.org.uk/IPG422
> Transcatheter aortic valve implantation for aortic stenosis – www.nice.org.uk/IPG421
> Endoscopic balloon dilatation for subglottic or tracheal stenosis – www.nice.org.uk/IPG425
> Focal therapy using cryoablation for localised stage prostate cancer – www.nice.org.uk/IPG423
> Focal therapy using high-intensity focused ultrasound (HIFU) for localised prostate cancer – www.nice.org.uk/IPG424

## Technology appraisals

> Venous thromboembolism – apixaban (hip and knee surgery) – www.nice.org.uk/TA245
